# Dissecting the Genetic Architecture of Host–Pathogen Specificity

**DOI:** 10.1371/journal.ppat.1001019

**Published:** 2010-08-05

**Authors:** Louis Lambrechts

**Affiliations:** Department of Virology, Institut Pasteur, Paris, France; The Fox Chase Cancer Center, United States of America

In this essay, I argue that unraveling the full genetic architecture (i.e., the number, position, effect, and interactions among genes underlying phenotypic variation) and molecular landscape of host–pathogen interactions can only be achieved by accounting for their genetic specificity. Indeed, the outcome of host–pathogen interactions often depends on the specific pairing of host and pathogen genotypes [1]. In such cases, the infection phenotype does not merely result from additive effects of host and pathogen genotypes, but also from a specific interaction between the two genomes (Box 1). This specific component, which can be measured by the interaction term in a two-way statistical analysis of phenotypic variation as a function of host and pathogen genotypes, is referred to as a genotype-by-genotype (G×G) interaction [1]. By analogy to genotype-by-environment (G×E) interactions that occur when different genotypes respond differently to environmental change, G×G interactions occur when the response of host genotypes differs across pathogen genotypes. Although the concept of G×G interactions has mostly been used by evolutionary ecologists to describe the specificity of host immune defenses against pathogens [2], it can be applied to any phenotype resulting from the specific interaction between two genomes. The general definition of G×G interactions allows its use to characterize phenotypes ranging from macroscopic traits such as lifespan [3] to the level of gene expression [4]. Here, the genetic specificity of host–pathogen associations is defined in the sense of G×G interactions. This definition differs from that of immunological specificity, which is the ability of a host to recognize and mount an immune response against a particular pathogen genotype or antigen. Whereas immunological specificity often depends on infection history (i.e., past exposure to a pathogen), genetic specificity describes the intrinsic compatibility between host and pathogen genotypes and occurs independently of infection history.

**Box 1. A Quantitative Genetic Model of Host–Pathogen Interactions**

Quantitative genetics is the area of genetics dealing with the inheritance of traits showing continuous phenotypic variation [35]. Typically, quantitative phenotypes are modeled as the result of combined effects of the genes (G) and the environment (E). The basic model to describe the phenotype of an individual is:
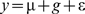
(Equation 1.1)where *y* is the phenotypic value of the individual, μ is the mean value of the population, *g* is the genetic contribution to the deviation from the mean (usually termed “genotypic value”), and ε is the environmental (non-genetic) deviation. By extending this model to a quantitative trait resulting from the interaction between a host and a pathogen, the model becomes:
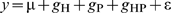
(Equation 1.2)where *g*_H_ is the host genotypic value, *g*_P_ is the pathogen genotypic value, and *g*_HP_ is the genotypic value due to the specific G×G interaction. This simple model ignores interactions between genes and environment (G×E and G×G×E effects), which occur when genotypic values vary across environments. The genetic component of phenotypic variance in a host–pathogen interaction can thus be partitioned into three distinct terms: variance due to the additive effect of the host genotype, variance due to the additive effect of the pathogen genotype, and variance due to the specific interaction between the two genomes. Whereas the first two terms can be characterized by considering either the host or the pathogen genetic variation alone, exploring the genetic basis of host–pathogen specificity requires that genetic variations in both the host and the pathogen are considered simultaneously.

In the case of a trait determined by two haploid loci *i* and *j* of a single organism, we can define α_i_ the additive effect of locus *i*, α_j_ the additive effect of locus *j*, and β_ij_ the interaction effect between loci *i* and *j* to decompose the genotypic value into:
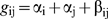
(Equation 2.1)Whereas non-additive interactions effects between different loci are defined under the term “epistasis”, when *i* and *j* are two homologous alleles of the same diploid locus the interaction effect is generally referred to as “dominance”. By defining Σα as the sum of all additive effects and Σβ as the sum of all interaction effects (both within and between loci), the previous equation can be generalized to any trait determined by multiple (n>2) loci:
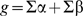
(Equation 2.2)By incorporating this expression into the general quantitative genetic model given by equation 1.1, we obtain the expression:
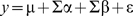
(Equation 1.3)Likewise, using the quantitative genetic model of host–pathogen interactions given by equation 1.2, it follows:
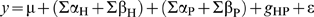
(Equation 1.4)By using the notations Σα_HP_ = Σα_H_+Σα_P_ (sum of additive effects of host and pathogen loci) and Σβ_HP_ = Σβ_H_+Σβ_P_+*g*_HP_ (sum of interaction effects between host loci, between pathogen loci, and specific G×G interactions between host and pathogen loci), the equation becomes:
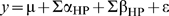
(Equation 1.5)The striking similarity between equations 1.5 and 1.3 illustrates how the phenotype of a host–pathogen interaction can simply be modeled as that of a third organism that combines both genomes. In such a model, the specific G×G interaction is included among all interaction terms, supporting the view that considering specificity in the genetic architecture of host–pathogen interactions is as important as including intra-genome epistasis. Like epistasis [36], [37], host–pathogen specificity may thus largely contribute to the unexplained genetic variation in susceptibility to infectious diseases missed by conventional QTL mapping strategies or genome-wide association studies [38], [39].

In some instances, the specificity of host–pathogen associations can be explained to a large extent by major genes of hosts and pathogens, as in the gene-for-gene model of plant–pathogen compatibility [5], [6]. In general, however, multiple genes and epistatic interactions among these genes determine the infection outcome [7]–[9]. A recent meta-analysis of 500 published studies reporting quantitative trait loci (QTL) for host resistance to pathogens in plants and animals revealed that the genetic architecture of this trait varies dramatically across different combinations of host and pathogen genotypes [9]. Thus, different host–pathogen associations involve different QTL and epistatic interactions, indicating that a substantial portion of phenotypic variation derives from the specific interaction between the two genomes. This is made even more complex when multiple pathogen species or strains infect the same host [10] and/or when G×G interactions are environment-dependent [11], [12].

It is striking that, to date, quantitative genetic studies of host–pathogen systems have neglected the specific component of the interaction. Dissecting the genetic architecture of complex infection traits has traditionally relied on QTL mapping strategies [7], [9] and more recently on association analyses of candidate gene polymorphisms [8]. A major caveat of these QTL mapping and association studies is that they focus on either the host or the pathogen genome. Because they consider variation in only one of the two interacting organisms, these studies ignore specific host genome by pathogen genome interactions. In order to fully dissect the genetic architecture and explore the molecular landscape of host–pathogen interactions, it will be necessary to account for the specific component of the relationship. This should be made possible by recent developments in molecular strategies combining host and pathogen genetics [13]–[15] and in quantitative genetic models of host–pathogen interactions allowing detection of host QTL by pathogen QTL interactions [16], [17]. Advantage could also be taken from existing methods for analysis of gene–gene and gene–environment interactions [18]–[21]. A critical (and limiting) aspect for investigating genetic specificity is the need to include different combinations of host and pathogen genotypes in the experimental design.

From a fundamental standpoint, improved knowledge of the genetic architecture of host–pathogen specificity has important implications for our understanding of the ecology and evolution of host–pathogen associations. The genetic specificity of host–pathogen interactions is thought to promote the maintenance of host and pathogen genetic diversity via frequency-dependent coevolutionary cycles [22]–[25], which in turn favor higher rates of mutation, recombination, and sexual reproduction [26]. Unraveling the genetic architecture and molecular landscape of host–pathogen specificity, combined with molecular evolution analyses, will shed light on the mechanistic basis of the infection process and the biochemistry of host–pathogen recognition [27]–[30]. The genetic model and precise epistatic interactions underlying host–pathogen specificity are critical determinants of coevolutionary dynamics and the evolution and maintenance of sex and recombination [27], [31]. In conjunction with gene flow and genetic drift, the genetic basis of specificity can also influence the spatial structure and local adaptation of host and pathogen populations [32].

From a more applied perspective, exploring the genetic basis of host–pathogen specificity will provide important insights into the mechanisms of disease emergence. Pathogens with a broad host range (i.e., a low degree of host specificity) are those most likely to emerge or re-emerge following ecological changes [33]. Disease emergence can also result from pathogen adaptation to a novel host species or population, which largely depends on the initial compatibility between host and pathogen genotypes [34]. Characterizing the genetic and molecular basis underlying host–pathogen specificity thus holds considerable promise for understanding, predicting, and preventing disease emergence. It will help to identify host species and populations most at risk for emergence of a given pathogen and uncover new molecular targets to interfere with the ability of emerging pathogens to jump from one host to another.

## References

[1] Lambrechts L, Fellous S, Koella JC (2006). Coevolutionary interactions between host and parasite genotypes.. Trends Parasitol.

[2] Schmid-Hempel P, Ebert D (2003). On the evolutionary ecology of specific immune defence.. Trends Ecol Evol.

[3] de Roode JC, Altizer S (2009). Host-parasite genetic interactions and virulence-transmission relationships in natural populations of Monarch butterflies.. Evolution.

[4] Riddell C, Adams S, Schmid-Hempel P, Mallon EB (2009). Differential expression of immune defences is associated with specific host-parasite interactions in insects.. PLoS ONE.

[5] Flor HH (1971). Current satus of the gene-for-gene concept.. Annu Rev Phytopathol.

[6] Thompson JN, Burdon JJ (1992). Gene-for-gene coevolution between plants and parasites.. Nature.

[7] Kover PX, Caicedo AL (2001). The genetic architecture of disease resistance in plants and the maintenance of recombination by parasites.. Mol Ecol.

[8] Lazzaro BP, Sceurman BK, Clark AG (2004). Genetic basis of natural variation in D. melanogaster antibacterial immunity.. Science.

[9] Wilfert L, Schmid-Hempel P (2008). The genetic architecture of susceptibility to parasites.. BMC Evol Biol.

[10] Wegner KM, Berenos C, Schmid-Hempel P (2009). Host genetic architecture in single and multiple infections.. J Evol Biol.

[11] Heath KD, Tiffin P (2007). Context dependence in the coevolution of plant and rhizobial mutualists.. Proc Biol Sci.

[12] Tetard-Jones C, Kertesz MA, Gallois P, Preziosi RF (2007). Genotype-by-genotype interactions modified by a third species in a plant-insect system.. Am Nat.

[13] Hill AV (2001). The genomics and genetics of human infectious disease susceptibility.. Annu Rev Genomics Hum Genet.

[14] Persson J, Vance RE (2007). Genetics-squared: combining host and pathogen genetics in the analysis of innate immunity and bacterial virulence.. Immunogenetics.

[15] Allen DE, Little TJ (2009). Exploring the molecular landscape of host-parasite coevolution.. Cold Spring Harb Symp Quant Biol.

[16] Wang Z, Hou W, Wu R (2006). A statistical model to analyse quantitative trait locus interactions for HIV dynamics from the virus and human genomes.. Stat Med.

[17] Yang J, Wu W, Zhu J (2008). Mapping interspecific genetic architecture in a host-parasite interaction system.. Genetics.

[18] Chanda P, Sucheston L, Liu S, Zhang A, Ramanathan M (2009). Information-theoretic gene-gene and gene-environment interaction analysis of quantitative traits.. BMC Genomics.

[19] Thomas D (2010). Methods for investigating gene-environment interactions in candidate pathway and genome-wide association studies.. Annu Rev Public Health.

[20] Kadarmideen HN, Li Y, Janss LL (2006). Gene-environment interactions in complex diseases: genetic models and methods for QTL mapping in multiple half-sib populations.. Genet Res.

[21] Demuth JP, Wade MJ (2006). Experimental methods for measuring gene interactions.. Annu Rev Ecol Evol Syst.

[22] Frank SA (1993). Coevolutionary genetics of plants and pathogens.. Evol Ecol.

[23] Little TJ, Watt K, Ebert D (2006). Parasite-host specificity: experimental studies on the basis of parasite adaptation.. Evolution.

[24] Sasaki A (2000). Host-parasite coevolution in a multilocus gene-for-gene system.. Proc Biol Sci.

[25] Tellier A, Brown JK (2007). Polymorphism in multilocus host parasite coevolutionary interactions.. Genetics.

[26] Hamilton WD, Axelrod R, Tanese R (1990). Sexual reproduction as an adaptation to resist parasites (a review).. Proc Natl Acad Sci U S A.

[27] Agrawal A, Lively CM (2002). Infection genetics: gene-for-gene versus matching-alleles models and all points in between.. Evol Ecol Res.

[28] Fenton A, Antonovics J, Brockhurst MA (2009). Inverse-gene-for-gene infection genetics and coevolutionary dynamics.. Am Nat.

[29] Frank SA (1994). Recognition and polymorphism in host-parasite genetics.. Philos Trans R Soc Lond B Biol Sci.

[30] Grosberg RK, Hart MW (2000). Mate selection and the evolution of highly polymorphic self/nonself recognition genes.. Science.

[31] Fenton A, Brockhurst MA (2007). Epistatic interactions alter dynamics of multilocus gene-for-gene coevolution.. PLoS ONE.

[32] Gandon S, Nuismer SL (2009). Interactions between genetic drift, gene flow, and selection mosaics drive parasite local adaptation.. Am Nat.

[33] Woolhouse ME, Gowtage-Sequeria S (2005). Host range and emerging and reemerging pathogens.. Emerg Infect Dis.

[34] Woolhouse ME, Haydon DT, Antia R (2005). Emerging pathogens: the epidemiology and evolution of species jumps.. Trends Ecol Evol.

[35] Lynch M, Walsh B (1998). Genetics and Analysis of Quantitative Traits.

[36] Brem RB, Storey JD, Whittle J, Kruglyak L (2005). Genetic interactions between polymorphisms that affect gene expression in yeast.. Nature.

[37] Kroymann J, Mitchell-Olds T (2005). Epistasis and balanced polymorphism influencing complex trait variation.. Nature.

[38] An P, Mukherjee O, Chanda P, Yao L, Engelman CD (2009). The challenge of detecting epistasis (G×G interactions): Genetic Analysis Workshop 16.. Genet Epidemiol.

[39] Thomas D (2010). Gene-environment-wide association studies: emerging approaches.. Nat Rev Genet.

